# Integrating values, ascribed responsibility and environmental concern to predict customers’ intention to visit green hotels: the mediating role of personal norm

**DOI:** 10.3389/fpsyg.2023.1340491

**Published:** 2024-01-08

**Authors:** Zheng Dong, Chang He, Tianyang Hu, Tianfeng Jiang

**Affiliations:** ^1^Business College, Qingdao University, Qingdao, China; ^2^School of Business, Shandong Jianzhu University, Jinan, China; ^3^Business School, University of Birmingham, Birmingham, United Kingdom; ^4^College of Business Administration, Lanzhou University of Finance and Economics, Lanzhou, China

**Keywords:** bipshperic values, altruistic values, egoistic values, ascribed responsibility, environmental concern, personal norms, tourism industry

## Abstract

**Introduction:**

It is crucial to understand the environmental friendly behavior of tourists. The utilization of natural resources by the tourists poses a significant threat to environmental sustainability. Fostering environmental friendly practices within tourism industry will help to protect future generation. The current study will evaluate the influence of values, ascribed responsibility, environmental concern and personal norms on customers’ intention to visit green hotels. Furthermore, it will assess the mediating effect of personal norms via ascribed responsibility and environmental concern.

**Methods:**

The study collected data from Data 347 customers through a questionnaire survey method. Partial least square-structural equation model (PLS-SEM) was employed for the analysis of data.

**Results and discussion:**

The findings indicate that values are significant factors affecting ascribed responsibility and environmental concern. Environmental concern direct impact on intention was insignificant. However, the environmental concern significantly affect intention to visit green hotels via personal norms indicating full mediating impact of personal norms.

## Introduction

In recent years, the tourism and hospitality sector has made a substantial contribution of approximately 10% to the global GDP (Hamid and Bano, 2022). Tourism brings several economic benefits such as the job creation, fostering growth in local economies, and contributing to revenue generation (Fan et al., 2019; Song et al., 2022). At the same time, deteriorating environmental health and environmental awareness are increasing customer interest in staying at green hotels (Waris et al., 2023). Wang et al. (2023) defined green hotel as “environmentally-friendly properties” whose managers are enthusiastic about implementing initiatives aimed at conserving water and energy, minimizing solid waste generation, and ultimately saving money, to protect the nature. Hotel management are currently placing increased emphasis on achieving a balance between environmental concerns and the utilization of resources (Wu and Cheng, 2019; Pan et al., 2022). Many hotels are developing green strategies in order to meet customers’ demand and address their environmental concerns. Pan et al. (2022) posit that hotels have reduced water usage through the implementation of high-efficiency plumbing fixtures. Additionally, they prioritize the use of building waste, sustainable wood and leather. Researchers indicated that implementing eco-friendly measures benefits both the hotel industry and the ecosystem (Eid et al., 2021).

Hotels across the globe have adopted a range of everyday green practices to address the growing demands of consumers and comply with the increasing environmental regulations (Agag and Colmekcioglu, 2020; Saleem, 2021). Consequently, hotel management have adopted the “green waves” by integrating green practices (Verma and Chandra, 2018; Chua and Han, 2022), and actively participate in green programs to demonstrate their commitment to environmental responsibility (Nimri et al., 2020). Recently, many researchers have argued that customers staying at green hotels represents pro-environmental behavior (PEB) (Peng et al., 2023). PEB refers to behavior aimed at minimizing harm to the environment (Steg and Vlek, 2009). Past research recognizes PEB as a diverse and multi-faceted concept that include behaviors in both private and public spheres (Ertz et al., 2016). The private sphere PEB include the purchase, utilization, and discarding of personal and household goods that have environmental consequences (Stern, 2000), including using cars and engaging in recycling practices (Ertz et al., 2023). It also includes conserving water and energy, minimizing the usage of solid waste, with the aim of protecting the environment (Wang et al., 2023). Conversely, public sphere PEB include behavior that directly impacts the environment through environmental activism, such as active participation in environmental organizations. It can also have an indirect effect by influencing public policies, such as petitioning on environmental issues (Stern, 2000). In recent years, there has been a growing popularity of green hotels in China (Yan and Chai, 2021; Wang et al., 2023). Despite the relatively new concept of green hotels, the number of such hotels in China has rapidly surpassed 700 (Wang, 2022). Nevertheless, several scholars have shown that the rise in the number of green hotels has not led to a substantial increase in booking revenues (D’Souza et al., 2020; Wang, 2022).

Extant literature depicts that there is a lack of research examining the behavior of customers in connection with green hotels. Nimri et al. (2020) reported that only 25.3% of the hospitality literature focuses on customers staying green hotels. The lack of research toward green hotels could be associated to the inconsistent findings observed in past studies. For example, some researchers reported that travelers willing to stay green hotels because of the perception that these hotels implementing environmental friendly practices (Rahman and Reynolds, 2016; Hasan, 2023). While other researchers reported that travelers prefer comfort and luxury during their stay at hotels (Shahid and Paul, 2022). Moreover, some travelers expressed reluctance to choose green hotels, expressing concerns about green practices of hotels, potentially viewing them as mere marketing strategies (Rahman and Reynolds, 2016). Another reason for the limited research in this area, as highlighted by Kim et al. (2017), could be the scarcity of literature related to the application of a theoretically grounded approach. Hence, more studies are required for a theoretically guided approach to assess and explain customers’ intention to visit green hotels.

Hotels customers in Asian countries, especially China (Wang, 2022), Bangladesh (Hasan, 2023), Pakistan (Sajjad et al., 2018), and India (Sadiq et al., 2022), have limited awareness and concerns regarding green hotels (Wang and Zhang, 2021). One of the potential reasons could be that the majority of previous research concerning pro-environmental behavior has been conducted in the western countries (Arun et al., 2021), depicting the consumers visit to green hotels is still, requiring a unified and systematic framework (Wang and Zhang, 2021). In response to immediate environmental problems, the Chinese government has undertaken various initiatives to increase community campaigns to share information regarding the threats due to unstainable practices in hotels (Wang et al., 2018). In addition, researchers have reported that customers’ values orientations (bioshperic values, altruistic values and egoistic values) have significant impact on intention to visit green hotels (Rahman and Reynolds, 2016; Fauzi et al., 2022; Wang et al., 2023). Values emphasis on an individual’s ability to engage in pro-environmental behavior (Wang et al., 2018), that extend beyond their immediate self-interest (Whitley et al., 2018). Recently, some studies have used values (biospheric, altruistic, egoistic) to effectively predict consumer visit to green hotels (Verma et al., 2019; Yan and Chai, 2021; Fauzi et al., 2022; Wang et al., 2023), and suggested that values have high predictive power in explaining pro-environmental behavior (Wang et al., 2022). However, there is a gap in understanding how values influences ascribed responsibility, environmental concern and personal norms toward customers’ green hotels visiting intention. Verma et al. (2019) evaluated the impact of values, ascription of responsibility, and environmental on attitude and green hotels visiting intentions. The authors call for more research on this area and exploring the relationships among the variables. Therefore, the current study will evaluate the influence of values (bioshperic, altruistic, and egoistic) on ascribed responsibility. Furthermore, the study will examine the influence of ascribed responsibility and environmental concern on personal norms and intention to visit green hotels which has been not examined in previous studies.

Hence, the current study aims to predict customers’ intention to visit green hotels through values (biospheric values, altruistic values and egoistic values), ascribed responsibility, environmental concern and personal norms. The values have the potential to exert sense of responsibility which in turn influences environmental concern and personal norms and eventually affect intention (Yan and Chai, 2021; Wang et al., 2023). Environmental concern reflects an enduring emotional state of an individual related to environment to issues (Hasan, 2023). Therefore, the current study added environmental concern as additional factor influencing the behavioral intention toward green hotels. There are three main contributions of the current study. First, the study will contribute to hospitality and tourism industry by examining the nexus between values, ascribed responsibility, and environmental concern toward customers’ intention to visit green hotels. Second, the study will assess the relationship between ascribed responsibility and environmental concern. Third, it will assess the direct and indirect impact of ascribed responsibility and environmental concern via personal norm on customers’ intention to visit green hotels. The findings of the study will provide insightful recommendations to the government and managers working in hospitality and tourism industry regarding customers’ intentions to visit green hotels.

## Literature review

### Values and pro-environmental behavior

It is described that individual decision making and green behavior are influences by values (Verma et al., 2019). Values play a crucial role in influencing the decision to engage (or not to engage) in pro-environmental behavior (De Groot and Steg, 2008). Past studies suggests that values can impact individual green behavior through both directly and indirectly. Individuals tend to be more responsive to outcomes that align with their values, increasing the acceptance of messages (Dietz et al., 1998). Values influence behavior by shaping beliefs and environmental concern, potentially influenced by selectively attending to information. The study conducted by Shin et al. (2017) posited that values function as a guiding framework for individual fostering a heightened awareness of environmental concerns. Additionally, Stern et al. (1993) suggested a three-dimensional value orientation consisting of egoistic, biospheric and altruistic values, which play a significant role in influencing (un)sustainable behavior. In previous studies, values were found to positively influence an individual’s environmental concern, norms, and attitude, consequently influencing their environmental behavior (Shin et al., 2017; Torkar and Bogner, 2019; Verma et al., 2019; Raza and Farrukh, 2023). Such relationships are widely accepted notion that values are convictions that function as benchmarks or standards for assessing actions, individuals, and occasions (Schwartz and Bilsky, 1990), therefore incorporated in the current study.

Although many past studies indicate that there is no difference between an altruistic and a biospheric value orientation (Stern et al., 1995; Nordlund and Garvill, 2002; Bardi and Schwartz, 2003; Choi et al., 2015). However, some empirical studies have examined biospheric and altruistic values independently (García Mira et al., 2003; Shin et al., 2017; Verma et al., 2019; Wang et al., 2023). For example, Stern et al. (1995) developed distinct scales for biospheric and altruistic values to explore the potential differences among three value constructs: biospheric, egoistic, and altruistic, which had not been empirically distinguished prior to their study. The findings revealed the differentiation among biospheric, altruistic, and egoistic value orientations, as determined by their validity and reliability. Therefore, it can be inferred that altruistic and biospheric values may be related, their interpretations vary in different environmental contexts.

## Hypotheses development

### Altruistic values

Altruistic values refer to individual motives to contribute for the welfare of people (Zasuwa, 2016). Altruistic values are important in shaping pro-environmental behavior (De Groot and Steg, 2008; Xu et al., 2022). Customers altruistic values appear to be more impartial and significantly influences environmental conservation through choice of green hotels (Tan et al., 2020). Van Riper and Kyle (2014) found positive effect altruistic values on individual responsibility and engagement in national park. According to Teng et al. (2014), altruism plays a crucial role in influencing customers to choose green hotels. The study conducted by Rahman and Reynolds (2016) indicated the people willingness to make sacrifices for the environment, while also exhibiting a favorable connection to their intention of visiting green hotels (Wang, 2022). Specifically, Verma et al. (2019) posits that individuals who exhibit strong altruistic values are inclined toward green hotels. Individual would only willingly make personal sacrifices for the environment if it benefits another person. Similarly, if individuals’ environmental concerns stem from altruistic values, they would act in accordance with moral principles and recognizes the value of other people and nature as an intrinsic end. Hence, based on these assumptions, it is hypothesized that:

*H1*: Altruistic values positively influences ascribed responsibility.

*H2*: Altruistic values positively influences environmental concern.

### Biospheric values

Biospheric values encompass core beliefs that reflect the concern for the well-being of the nautre (Stern et al., 1993). It emphasizes individual focus on the environmental quality (De Groot and Steg, 2008). Individual who exhibit high biospheric values prioritize nature and make decisions by considering the costs and benefits to the ecosystem (Li et al., 2021). Past studies indicate that biospheric values play an important role in influencing decisions regarding environmentally friendly behavior (Katz-Gerro et al., 2017; Kim and Seock, 2019). Biospheric values seem to encompass various motivations for environmentally friendly behavior, making it a significant factor affecting individual responsibility toward the environment (Verma et al., 2019). Based on the findings of the past studies, this study assumes that biospheric values will positively influence customers’ ascription of responsibility and environmental concern. Hence, it is hypothesized that:

*H3*: Biospheric values positively influences ascribed responsibility.

*H4*: Biospheric values positively influences environmental concern.

### Egoistic values

The egoistic values refer to an individual’s tendency prioritizing a favorable balance between cost and benefits for their own benefits (Liu et al., 2018). Egoistic individual participates in environmental preservation solely when they perceive a direct threat to their personal well-being (Van Riper and Kyle, 2014). Moreover, previous studies indicate that egoistic values have a positive impact on environmental behavior (Stern et al., 1995; Arya and Kumar, 2023). This is attributed to individuals favoring a specific environmental standard due to the anticipated personal benefits, leading to an increase in utility. Egoistic values reflect an individual emphasizes on self-improvement. Individual egoistic value orientation consciously evaluate specific actions based on their perceived costs and benefits. For instance, Ojea and Loureiro (2007) revealed strong and positive correlation between egoistic value orientation and the willingness to contribute financially to wildlife conservation efforts. Kim and Seock (2019) found that egoistic values has positive and significant impact on individual purchase of green apparel. Verma et al. (2019) indicate that an individual with a strong egoistic orientation will select the green hotels if the perceived benefits outweigh the perceived costs. The current study assumes that egoistic value will have a positive influence on ascribed responsibility and environmental concern. Hence, it is hypothesized that:

*H5*: Egoistic values will positively influence ascribed responsibility.

*H6*: Egoistic values will positively influence environmental concern.

### Ascribed responsibility

Ascribed responsibility refers to the sense of accountability individuals experience for the adverse outcomes resulting from their failure to engage in pro-environmental behavior (Raza and Farrukh, 2023). Ascribed responsibility of individuals plays a crucial role in shaping their environmental behaviors. This study refers ascribed responsibility as the sense of responsibility for the adverse outcomes resulting from a lack of personal obligation toward the environment. Individual tend to exhibit more environmentally friendly behavior when they have an understanding of their adverse effects on the environment and share a sense of mutual responsibility for the subsequent negative consequences. On the contrary, the strength of the relationship weakens when individuals possess a minimal sense of assigned responsibility (De Groot and Steg, 2009; Verma et al., 2019). From the hotel perspective, individual consciousness of environmental adverse effects is likely to impact the choice of staying at a green hotel (Han et al., 2015). Individuals who believe they bear responsibility for environmental issues resulting from their actions are more inclined to participate in environmentally friendly behavior (Nyborg et al., 2006). Several past studies also confirmed the significance of ascribed responsibility as a predictor of ecological behaviors (Hines et al., 1987; Granzin and Olsen, 1991; Verma et al., 2019; Raza and Farrukh, 2023).

Numerous studies have explored the role of personal norms as mediator when examining the impact of ascribed responsibility on behavioral intentions (Jakovcevic and Steg, 2013; Van Riper and Kyle, 2014; Han, 2015; Kim and Seock, 2019; Verma et al., 2019). Nevertheless, none of these studies have examined ascribed responsibility as a direct predictor to personal norm and environmental concern, factors that may subsequently influence pro-environmental behavior. The current study will evaluate the direct and indirect impact of ascribed responsibility on intention to visit green hotels. Hence, it is hypothesized that:

*H7*: Ascribed responsibility will positively influence environmental concern.

*H8*: Ascribed responsibility will positively influence personal norms.

*H9*: Ascribed responsibility will positively influence intention to visit green hotels.

### Environmental concern

Environmental concern refers to individual awareness of environmental issues and their willingness to personally contribute to solutions (Hu et al., 2010). It refers to the extent to which individuals are aware of environmental issues and express a readiness to personally contribute to their resolution (Peisker, 2023). Researchers have recognized environmental concern as an important antecedent of pro environmental behavior (Chen and Hung, 2016; Verma et al., 2019). It is the collection of individual beliefs regarding the environment and the relationship between humans and their environment (Wu et al., 2022). Past studies indicate that an individual concern regarding environmental matters is a significant element of environmental behaviors, ranging from conserving energy and recycling waste to engaging in green purchasing (Manaktola and Jauhari, 2007; Hu et al., 2010; Verma et al., 2019).

Furthermore, past studies consistently demonstrate that individual personal norms toward eco-friendly products and services is significantly influenced by their environmental concerns (Yadav and Pathak, 2016; Wu et al., 2022). For example, the study conducted by Wu et al. (2022) revealed positive impact of personal norms on students’ waste management behavior. Koklic et al. (2019) conducted study on the purchase on organic foods indicate the positive influence of environmental concern on personal norms. There, this study assumes that environmental concern will positively influence on personal norms regarding customers’ intention to visit green hotels. Hence, it is hypothesized that:

*H10*: Environmental concern will positively influence customers’ personal norms.

*H11*: Environmental concern will positively influence intention to visit green hotels.

### Personal norm as a mediator

**Figure 1:**
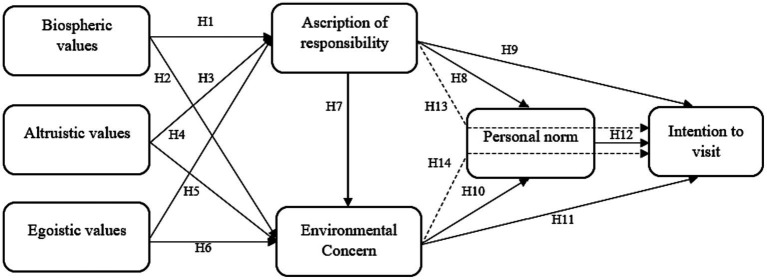
Conceptual framework. Dotted lines represents the mediating relationships.

Personal norm refers to an individual’s sense of moral duty toward engaging in a particular behavior (Schwartz, 1977). Researchers determined two pre-requisite conditions for the activation of personal norms. Firstly, individuals must possess an awareness that refraining from engaging in pro-social behavior can result in negative outcomes for others. Secondly, they must acknowledge their own responsibility for these adverse consequences (Wang et al., 2018). Researchers reported that pro-environmental behaviors as an act of altruism aimed at enhancing the welfare of human being (Stern et al., 1993; Wang et al., 2018; Wu et al., 2022). In past research found that personal norms play a significant role in shaping individual behavioral intention to visit green hotels (Fauzi et al., 2022; Wang et al., 2022, 2023). Additionally, studies have reported that personal norm is a significant mediator in individual pro-environmental behavior (Wang et al., 2018; Kim and Seock, 2019; Ateş, 2020). Wang et al. (2018) study’s findings indicate that personal norm is a significant mediator between information publicity and intention to recycle e-waste. Kim and Seock (2019) revealed that personal norm significantly mediates the relationship between social norm and purchasing of environmentally-friendly apparel. Ateş (2020) found mediating impact of personal norm between environmental self-identity and pro-environmental behavior. Past studies confirmed the mediating impact of personal norm in pro-environmental behavior (Figure 1). Hence, it is hypothesized that:

*H12*: Personal norms will positively influence customers’ intention to visit green hotels.

*H13*: Personal norms will positively mediate the relationship between ascribed responsibility and customers’ intention to visit green hotels.

*H14*: Personal norms will positively mediate the relationship between environmental concern and customers’ intention to visit green hotels.

## Methodology

### Instrument

The respondent data was collected using a self-administered questionnaire. The current study adopted measurement scales from past studies to measure intention, personal norms, environmental concern, ascribed responsibility and values (altruistic, biospheric, and egoistic) in the context of visiting green hotels. The measuring items for altruistic and egoistic constructs were adapted from Shin et al. (2017) study. The authors reported 0.83 and 0.89 composite reliability (CR) for altruistic and egoistic values, respectively. The measuring items for biospheric value were adapted from Sharma and Gupta (2020) study with the CR value above 0.70. The measuring items for the ascribed responsibility were adapted from Choi et al. (2015) and Raza and Farrukh (2023) studies with the CR values of 0.928 and 0.881, respectively. The measuring items for the environmental concern were adapted from the Verma et al. (2019) study with the CR value 0.845. The measuring items for personal norm were adapted from Choi et al. (2015) study with the CR value of 0.910. The measuring items for the intention to visit green hotels were adapted from the Verma et al. (2019) with CR value of 0.899. The constructs validity was assessed through factor loadings and average variance extracted (AVE) values. In past studies, constructs factor loadings were greater than 0.70, and AVE values were greater than 0.50 confirming convergent validity. The measuring items were evaluated using a 5-point Likert scale that ranged from ‘strongly disagree’ at 1 to ‘strongly agree’ at 5. The details regarding the constructs and items used in the study, as well as their sources, are provided in Table 1. Although the questionnaire has been implemented in India and Pakistan, which have collectivistic culture similar to China, it has been back translated to remove ambiguities. The questionnaire contained 25 measuring items. A pilot study was conducted by administering a total of 45 questionnaires to the hotel customers. Based on the results of the pilot test, the personal norm item PN5, “I personally feel a responsibility to conserve energy to the greatest extent possible,” was removed due to its low factor loading. Finally, the purposive sampling technique was employed to distribute a total of 505 questionnaires among hotel customers. Though the findings derived from the purposive sampling technique may not be generalizable, they still justify the appropriateness of conducting the study on a particular population (Cheah and Phau, 2011; Verma et al., 2019), but it enables the researchers to control the sample’s representativeness through self-judgement to meet the research objectives (Waris et al., 2023). The study specifically selects participants who visited hotels for at least 1 day. These participants were purposively selected to ensure to meet the research objectives. Further, following the Kline’s (1998) suggestion, 240 (24 items) sample size was enough for data analysis. However, we distributed the questionnaire to 505 to increase reliability. At the end of data collection, we received 347 usable sample from hotel customers.

**Table 1:** Constructs’ items and sources.

Construct	Items	Sources
Altruistic values	AL1: I appreciate a world without war and conflict.	Shin et al. (2017)	AL2: I like to correct injustice.		AL3: I have a concern for those who are vulnerable and elderly.	
Bishopheric values	BV1: I am committed to the prevention of pollution and the preservation of natural resources.	Sharma and Gupta (2020)	BV2: I advocate for accepting and honoring nature instead of trying to exert control over it.		BV3: I believe in protecting environment and the conservation of nature.	
Egoistic values	EGV1: I respect social power.	Shin et al. (2017)	EGV2: I value wealth.		EGV3: I hold social recognition in high regard.	
Ascribed responsibility	ASR1: I feel the shared responsibility for the problems related to energy.	Choi et al. (2015) and Raza and Farrukh (2023)	ASR2: I feel shared responsibility for the depletion of energy resources.		ASR3: I believe I share a collective responsibility for the global warming.		ASR4: I have contributed to reducing energy problems.	
Environmental concern	EC1: The balance of nature is very delicate and can be easily disturbed.	Verma et al. (2019)	EC2: Human beings are severely abusing the environment.		EC3: Human being must maintain the balance with nature to survive.		EC4: Human interferences with nature often produce disastrous consequences.	
Personal norm	PN1: I believe that I would be a better human by choosing to stay at green hotels and utilizing green products and services.	Choi et al. (2015)	PN2: I sense a moral obligation to choose a green hotel over a conventional one.		PN3: I feel responsible to consider the environment and nature in my traveling.		PN4: I feel morally obliged to choose green hotels, irrespective of the choices made by others		PN5: I personally feel a responsibility to conserve energy to the greatest extent possible*.	
Intention	INT1: I am willing to stay at a green hotel when I travel.	Verma et al. (2019)	INT2: I plan to stay at a green hotel when I travel.		INT3: I will make an effort to stay at a green hotel when I travel.	

* Deleted item due to low factor loading.

### Data collection

To ensure the data collection from the large number of tourists, the questionnaires were distributed to all customers who have visited hotels once in a life. The data was collected from two major cities in Chian Beijing and Tianjin. These cities are globally renowned therefore customers around the world visits these cities and explore the country. In addition, tourists are highly inclined to engage in tourist activities during the Chinese Spring Festival, which is the lengthiest holiday in China occurring between January and March. This enabled the collection of data in a more convenient manner, reflects the population of the research. Out of the 505 questionnaires distributed to respondents, a total of 347 usable questionnaires were returned, 22 questionnaires were incomplete and, as a result, have been discarded. Further, we performed mahalanobis distance technique and removed 7 outliers from the data. The response rate was 68.71%. Finally, the analysis was performed using 347 sample data. The details of the study’s participants are given in Table 2.

**Table 2:** Demographic profile.

		Frequency	Percentage
Gender	Men	201	57.9	Female	146	42.1
Age in years	18 to 30	94	27.1	31 to 40	105	30.3	41 to 50	66	19.0	51 to 60	53	15.3	61 and above	29	8.4
Qualification	High School and Diploma	9	2.6	Bachelor	79	22.8	Masters and MPhil	168	48.4	PhD	91	26.2
Monthly Income in CNY	≤ 7,000	154	44.4	7,001 to 10,000	78	22.5	10,001 to 12,000	33	9.5	12,001 to 15,000	17	4.9	15,001 to 18,000	18	5.2	> 18,000	47	13.5

CNY, Chinese Yuan.

## Results

This study aims to analyze the relationships within the proposed model, aiming to explore influence of values (altruistic, bioshperic and egoistic values) on ascribed responsibility. Further, the study assesses the impact of ascribed responsibility and environmental concern on personal norm and intention to visit green hotels. To achieve this objective, the study employed the partial least squares-structural equation modeling (PLS-SEM) technique using SmartPLS version 4.0.9.8. To test the mediating relationships PLS-SEM is considered an appropriate tool for analysis (Hair et al., 2014). According to Dash and Paul (2021) offers several advantage when assessing a mediating relationship, particularly assessing simultaneous relationships in a model. Hair et al. (2014) suggested to perform two stage processes when testing hypothesis with PLS-SEM. First stage involves assessing validity and reliability using the measurement model, and second stage involves hypothesis testing through the bootstrapping procedure, which includes generating 5,000 samples.

### Common method bias (CBM)

Before assessing measurement model and structural model we evaluated data for the presence of common method bias (CBM), which is regarded as a potential issue that inflate study’s findings (Fuller et al., 2016). We conducted a factor analysis wherein all variables were included, and the extraction of factors was restricted to a single factor. If a single factor can explain over 50% of the variance, this shows a potential threat to the data credibility. The result of single factor analysis revealed 26.23% variance, below the specified threshold. Therefore, we can conclude that CMB is not an issue for current study.

### Measurement model

The analysis of measurement model involves an assessment of convergent validity, reliability, and discriminant validity of all constructs. Factor loadings and Average variance extracted (AVE) were assessed to determine convergent validity. The convergent validity establishes when the factor loadings should be equal to or exceed 0.70, and the AVE values should be 0.50 or higher. Reliability was assessed using Cronbach’s alpha and composite reliability (CR), with the criterion of achieving values equal to or exceeding 0.70 (Hair et al., 2019). Personal norm item (PN5) was below 0.70 was eliminated from the analysis (Hair et al., 2017). Table 3 illustrates the convergence validity and reliability, demonstrating that the hypothesized model meets the requirements of PLS-SEM.

**Table 3:** Measurement model.

Construct	Items	Factor loading	Cronbach’s alpha (α)	Composite reliability (CR)	Average variance extracted (AVE)
Altruistic value	AL1	0.839	0.824	0.895	0.740	AL2	0.888				AL3	0.844			
Biospheric value	BV1	0.851	0.798	0.881	0.712	BV2	0.836				BV3	0.847			
Egoistic value	EGV1	0.879	0.846	0.906	0.763	EGV2	0.854				EGV3	0.847			
Ascribed responsibility	ASR1	0.844	0.852	0.900	0.692	ASR2	0.793				ASR3	0.846				ASR4	0.842			
Environmental concern	EC1	0.867	0.878	0.916	0.732	EC2	0.880				EC3	0.846				EC4	0.819			
Personal norm	PN1	0.755	0.805	0.871	0.628	PN2	0.800				PN3	0.783				PN4	0.835			
Intention	INT1	0.903	0.825	0.896	0.741	INT2	0.842				INT3	0.837			

To verify the validity of the measurement model, this study employed two criterions for assessing the discriminant validity: Fornell and Larcker (1981) and heterotrait–monotrait ratio (HTMT). The first criterion involved checking the square root of the AVE for each construct to ensure it exceeded its highest correlation with any other construct (Fornell and Larcker, 1981). The second criterion employed is HTMT. Henseler et al. (2015) have argued that the HTMT represents a better criterion for evaluating discriminant validity. The HTMT ratio is determined by comparing the average correlations of indicators across constructs that assess various facets of the model with the average correlations of indicators within the same construct. Values less than 0.85 suggest satisfactory discriminant validity. The results indicate in Table 4 and Table 5 confirming discriminant validity via Fornell and Larcker (1981) and HTMT criterions, respectively.

**Table 4:** Fornell and larcker criterion.

	1	2	3	4	5	6	7
AL	**0.859**						
ASR	0.301	**0.832**					
BV	0.241	0.250	**0.844**				
EC	0.299	0.189	0.210	**0.855**			
EGV	0.224	0.564	0.209	0.159	**0.873**		
INT	0.374	0.503	0.190	0.229	0.522	**0.861**	
PN	0.260	0.339	0.457	0.325	0.308	0.336	**0.793**

The bold diagonal values refer to the square root of the AVE of each construct. All correlations are statistically significant (*p* < 0.01).

**Table 5:** Discriminant validity Heterotrait-monotrait ratio (HTMT).

	1	2	3	4	5	6	7
Altruism
Ascribed responsibility	0.355						
Biospheric value	0.292	0.297					
Environmental concern	0.356	0.216	0.253				
Egoistic value	0.257	0.647	0.249	0.180			
Intention	0.448	0.591	0.232	0.265	0.620		
Personal norm	0.305	0.400	0.574	0.368	0.363	0.398	

### Structural model

The structural model examination involves the significance of path coefficients (beta), coefficient of determination (R2), the effect size (f2), and predictive relevance (Q2). First, the study assessed variance inflation factor (VIF) determine presence of multi-collinearity (Hair et al., 2011). The VIF coefficients below 3.3 indicate that the model is free from both vertical and lateral collinearity (Kock, 2015). The study’s findings show that the VIF values for the independent variables are below the specified threshold of 3.3 (Hair et al., 2010). This indicates that collinearity is not a significant concern in the subsequent structural model. The current study also considered the effect size (*f*^2^). Cohen (1988) categorized that independent variables as having a small, medium, and large effect for (*f*^2^) values of 0.02, 0.15, and 0, respectively. Table 6 indicate that AL and BV have small effect on ASR (*f*^2^ = 0.038; 0.016). However, EGV has large effect on ASR (*f*^2^ = 0.369). ASR has small effect on EC (*f*^2^ = 0.037). The effect size of ASR and EC on PN is also small (*f*^2^ = 0.098; 0.087). EC and PN have small effect on INT (*f*^2^ = 0.012; 0.029). However, the ASR has large effect on INT (*f*^2^ = 0.231).

**Table 6:** Effect size (f^2^) results.

	1	2	3	4	5	6	7
AL		0.039		0.011			
ASR				0.122		0.138	0.037
BV		0.018		0.011			
EC						0.015	0.045
EGV		0.347		0.077			
INT							
PN						0.032	

The predictive relevance of the model was evaluated by employing the Stone–Geisser (Q^2^) coefficients employing the blindfolding process with a distance of 7 (Li et al., 2023). The Q^2^ value for the endogenous greater than 0 indicate acceptable predictive relevance (Hair et al., 2017). Then, the study employed 5,000 resamples bootstrapping technique to generate path coefficients and t-values. This was done to examine the hypothesized relationships among variables. Cohen (1988) suggested that the interpretation of R2 can be categorized as weak, moderate and substantial. The *R*^2^ value between 0.02 and 0.12 is weak, 0.13 and 0.25 is moderate, and above 0.26 is substantial. In this study, the *R*^2^ value for intention to visit green hotels is 29.3%, indicate that independent and mediating variables explain 29.3% variance. As indicated in Table 7, the path coefficients were significantly positive at a 95% level of confidence: AL to ASR (*β* = 0.163, *p* < 0.05), BV to ASR (*β* = 0.105, *p* < 0.05), and EGV to ASR (*β* = 0.505, *p* < 0.05). ASR to EC was positively significant (*β* = 0.189, *p* < 0.05); ASR to PN was positively significant (*β* = 0.287, *p* < 0.05), and ASR to INT was positively significant (*β* = 0.432, *p* < 0.05). EC to PN was positively significant (*β* = 0.271, *p* < 0.05) and EC to INT was insignificant (*β* = 0.125, *p* > 0.05). PN to INT positively significant (*β* = 0.158, *p* < 0.05). The results for the direct relationship male group was consistent with the overall sample except the positive influence of BV on ASR (*β* = 0.118, *p* > 0.05). However, for the female group, the positive influence of BV on ASR (*β* = 0.094, *p* > 0.05), BV on EC (*β* = 0.025, *p* > 0.05), EC on INT (*β* = 0.071, *p* > 0.05), and PN on INT (*β* = 0.210, *p* > 0.05) were insignificant.

**Table 7:** Hypotheses testing.

Complete					Male				Female			
Hypotheses	Beta	*p*	T	Results	Beta	*p*	T	Results	Beta	*p*	T	Results
H1: AL - > ASR	0.167	0.001	3.323	Supported	0.290	4.540	0.000	Supported	0.033	0.462	0.644	Supported
H2: AL - > EC	0.090	0.032	2.139	Supported	0.147	2.181	0.029	Supported	0.058	0.796	0.426	Supported
H3: BV - > ASR	0.111	0.016	2.413	Supported	0.118	1.767	0.077	Not Supported	0.094	1.238	0.216	Not Supported
H4: BV - > EC	0.087	0.031	2.162	Supported	0.125	2.225	0.026	Supported	0.025	0.361	0.718	Not Supported
H3: EGV - > ASR	0.493	0.000	9.971	Supported	0.442	6.897	0.000	Supported	0.539	7.349	0.000	Supported
H6: EGV - > EC	0.266	0.000	4.921	Supported	0.251	3.960	0.000	Supported	0.289	3.125	0.002	Supported
H7: ASR - > EC	0.345	0.000	5.982	Supported	0.258	2.689	0.007	Supported	0.413	5.726	0.000	Supported
H8: ASR - > PN	0.210	0.000	3.082	Supported	0.262	2.983	0.003	Supported	0.208	2.053	0.040	Supported
H9: ASR - > INT	0.377	0.000	6.466	Supported	0.360	4.994	0.000	Supported	0.396	3.935	0.000	Supported
H10: EC - > PN	0.233	0.001	3.325	Supported	0.239	2.524	0.012	Supported	0.274	2.689	0.007	Supported
H11: EC - > INT	0.125	0.021	2.301	Supported	0.146	2.105	0.035	Supported	0.071	0.773	0.439	Not Supported
H12: PN - > INT	0.164	0.004	2.854	Supported	0.166	2.255	0.024	Supported	0.210	2.456	0.014	Not Supported
H13: ASR - > PN - > INT	0.038	1.939	0.053	Partial Mediation	0.043	1.811	0.070	No mediation	0.044	1.364	0.173	No mediation
H14: EC - > PN - > INT	0.035	2.010	0.044	Partial Mediation	0.040	1.446	0.148	No mediation	0.058	1.799	0.072	No mediation

The study also examined the mediating effect in accordance with Hayes and Preacher’s (2014) recommendations, assessing the mediating influence of personal norm to visit green hotels. They suggested that if the confidence interval does not include zero (0), it represents the mediating effect. The mediating effect of PN was significant. Hence, the study conclude that PN partially mediates the relationships between ASR and INT (*β* = 0.038, *t* = 1.939 *p* < 0.10) and EC and INT (*β* = 0.035, *t* = 2.010, *p* > 0.05). However, the mediating results for male and female groups indicate no mediating effect of PN on the relationships between the ASR and INT and EC and INT. The results are summarized in Table 7.

### Multi-group analysis

**Figure 2:**
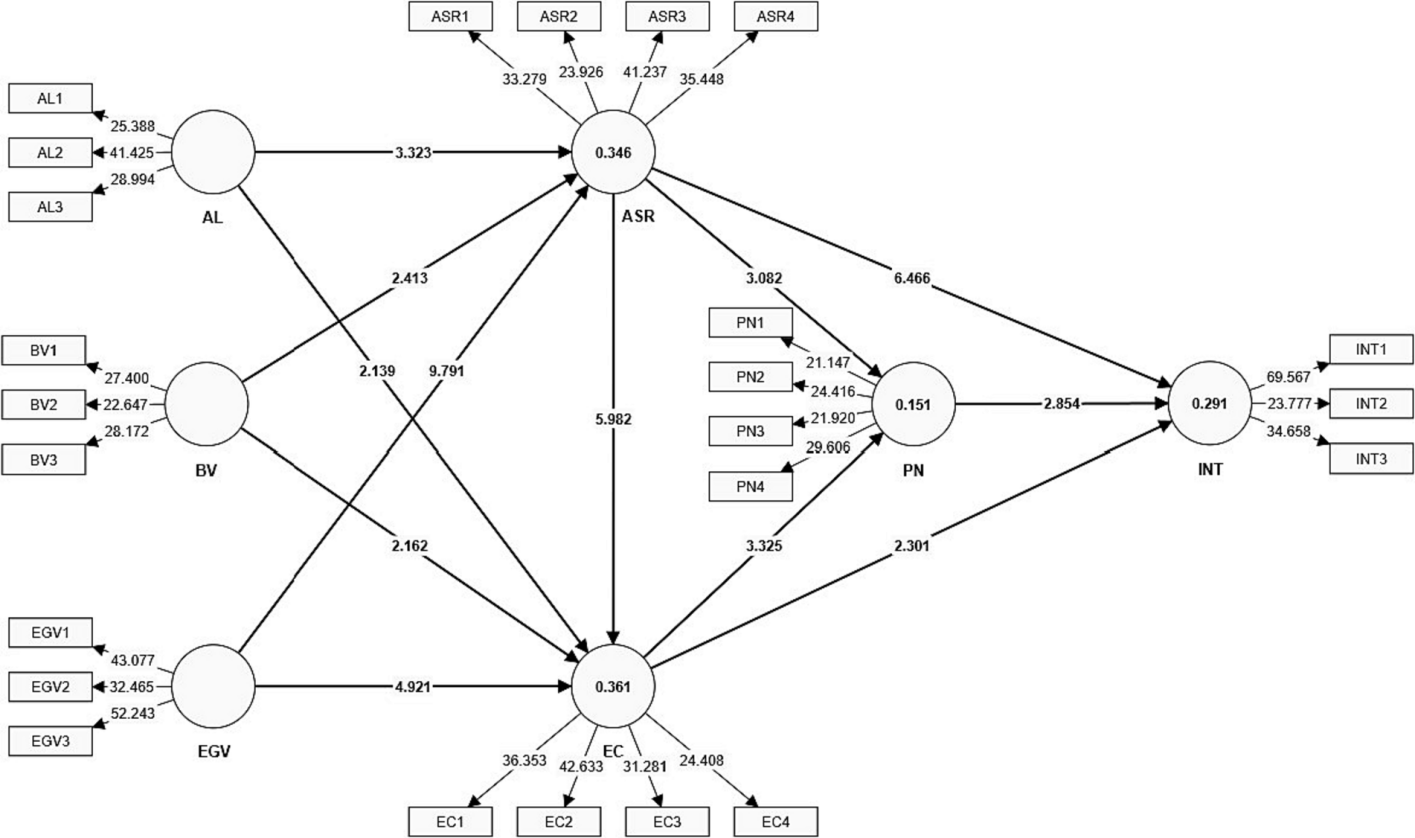
Strucural mo.

In the concluding part of the results analysis, we examined the difference between male and female in terms of direct relationships. The results of multi-group analysis indicate that all differences were found to be insignificant, except in the relationship between AL and ASR. The results indicate that impact of AL on ASR was stronger in male in comparison to female. The findings from the multi-group analysis are presented in Table 8 and Figure 2.

**Table 8:** Multi-group analysis.

Relationships	Difference (Male - Female)	*p* value
AL - > ASR	0.258	0.003
AL - > EC	0.089	0.180
BV - > ASR	0.024	0.410
BV - > EC	0.100	0.127
EGV - > ASR	−0.096	0.162
EGV - > EC	−0.038	0.363
ASR - > EC	−0.154	0.099
ASR - > PN	0.054	0.347
ASR - > INT	−0.036	0.382
EC - > PN	−0.034	0.404
EC - > INT	0.074	0.261
PN - > INT	−0.045	0.344

## Discussions and conclusion

The objective of the study was to assess the impact of values (altruistic, biospheric, and egoistic) on ascribed responsibility. Furthermore, the study evaluated the direct and indirect impact of ascribed responsibility and environmental concern on intention to visit green hotels via personal norm. The findings of the study revealed that proposed conceptual model effectiveness to predict customers’ intention to visit green hotels. The results indicate that values (altruistic, biospheric, and egoistic), ascribed responsibility are essential element predicting intention to visit green hotels. The findings are consistent with previous studies (Han, 2015; Verma et al., 2019; Wang et al., 2023) where the authors demonstrated that the values (altruistic, biospheric, and egoistic) regarding the environment can influence customers’ intention to visit green hotels. Additionally, it is recognized that green hotels tend to be more expensive. For customers to find value in the green hotels, services must be higher than the premium price.

The findings of the study show that altruistic values have significant ascribed responsibility and environmental concern depicting that hotel customers are selfless and responsible, as they consider visiting green hotels to be the right thing to do. The findings are consistent with Roos and Hahn (2019), Van Riper and Kyle (2014) and Han et al. (2015). The positive influence of biospheric values on ascribed responsibility and environmental concern is in line with the studies of Choi et al. (2015) and Verma et al. (2019), where authors emphasized the significant role of biospheric values on intention to visit green hotels. These findings indicate that customers are willing to take measures to protect environment. These customers value environmental sustainability and willing to visit green hotels that implement green practices. The study’s findings also show a positive and significant influence of egoistic value on ascribed responsibility and environmental concern. The findings are consistent to Kim and Seock (2019) and Verma et al. (2019) studies. Researchers have confirmed that individuals driven by self-interest exhibit environmentally friendly behavior when they perceive personal advantages in the outcome of such behavior. In the context of the current study, the results indicate that customers who perceive personal benefits from visiting green hotels are more likely to develop sense of responsibility and care for the environment. Further, the findings indicate the positive significant impact of ascribed responsibility on environmental concern which is in line with Verma et al. (2019) findings. The positive relationship between ascribed responsibility and environmental concern depict that customers’ have sense of responsibility toward protecting environment. The positive influence of ascribed responsibility on personal norm is also confirmed which is consistent with Choi et al. (2015) study’s findings. People who consider that visiting green hotels will align with their values have tendency to visit green hotels. The positive influence of environmental concern on personal norm is consistent with Koklic et al. (2019) study’s findings. This shows that customers care for the environment is ingrained in their value system that influences personal norm. The positive influence of environmental concern on intention to visit green hotels is insignificant. The results match with Teng et al. (2014) study findings that argued that environmental concern does not affect individual intention to visit green hotels. The positive significant impact of personal norm on intention to visit green hotel matches with previous studies (Fauzi et al., 2022; Wang et al., 2022, 2023). The findings also confirmed positive influence of environmental concern on intention via personal norm confirming the full mediating effect. In addition, the study confirmed the mediating impact of personal norm on the relationship between ascribed responsibility and intention to visit green hotels, signifying the importance of individual sense of responsibility to contribute toward environmental preservation by staying at green hotels. The findings indicate that customers with strong personal beliefs to protect the environment likely to patronize green hotels in the future.

## Implications

### Theoretical implications

This study adds to body of knowledge in the field of hospitality in several ways. First, the study seeks to examine the customers’ intention to visit green hotels. The current study contributes to the literature of hospitality and tourism by establishing the relationship between values, ascribed responsibility and environmental concern. Furthermore, the current study model is unique as it adds personal norm as mediating factor between environmental concern and intention. Past studies have neglected this relationship. The previous research has predominantly used Ajzen’s (1991) TPB model to predict customers’ intention to visit green hotels (Verma and Chandra, 2018; Pan et al., 2022). The current study developed a model that integrate the values in a chain relationship with personal norm and intention making it a novel theoretical addition. Most of the results confirmed Stern’s VBN theory, indicating that individuals with strong altruistic, biospheric and egoistic values display strong pro-environmental beliefs. Consequently, this can foster strong personal norms that encourage environmental behavior. Individuals possessing strong personal norm display greater intention to visit a green hotel compared to those lacking in personal norm. Additionally, the current study established full mediating influence of personal norm in the relationships between environmental concern and intention to visit green hotels. This reflect that personal norm is a crucial factor in that exerts significant influence on individual environmental behavior.

### Practical implications

The current study has multiple practical implications for the practitioners and managers of green hotels. Based on the study findings, hotels aiming to enhance customers’ intention to visit green hotels should focus on their green offerings in alignment with customers’ values and personal norms. As highlighted by van Riper and Kyle (2014), the focus is not on the impact of values on consumers’ behavioral outcomes, but rather on how these variables can be translated into action. Therefore, it is necessary for the hotels’ management to use their marketing efforts to convey their commitment to environmentally friendly practices to customers. For instance, hotels can educate the potential customers regarding responsibility and environmental concern that improve environmental sustainability. Therefore, it is recommended that hotels managers should enhance the frequency of disseminating eco-friendly information and broaden the promotional campaigns reach. Furthermore, it is evident from the findings that tourism industry marketers need to focus on increasing individuals’ values and environmental concern that will ultimately lead to the development of personal norms supporting green hotels. For example, the advertisement for the green hotels employ a compelling message like “the majority of people recycle their waste” rather than solely stating “please recycle” (Verma et al., 2019). To enhance the efficiency of hotel promotions for environmental conservation, an effective approach is to leverage consumer emotions. Judah et al. (2009) propose utilizing a technique of public shaming to further instill a sense of responsibility in their actions. In addition, to increase the visit of green hotels, it is recommended to conveying the message in the hotel lobby “Is the person next to you choose green hotels while traveling?” In line with this, we suggest that hotel managers who aim to decrease water or electricity consumption in guest rooms could also consider exhibiting these messages at the reception area. The findings of this research offer a necessary basis for policymakers and businesses to take action in influencing the personal norms of customers’ who may be unaware or indifferent to environmental harm caused by hotels unsustainable practices. Therefore, the establishment of green hotels, driven either by customers’ demand or external influences, will contribute to the development toward a more environmentally conscious and sustainable society. This research contributes to the literature of hospitality by assessing the novel connections that lead to intention and provide valuable insights to managers to understand customers’ behavior.

### Limitations and future research scope

Although the current study contributes to the literature of hospitality by developing a novel framework, it has also some limitations that pave the way for future researches. The generalizing of the current study results may be limited because the data collected from customers in China. The representativeness of population could have been enhanced by randomly selecting customers from various cities in China. Additionally, this study primarily evaluated consumers’ intention to visit green hotels and did not consider their actual visiting behavior. As a result, it creates an opportunity for future research to gather data over a period of time and examine the actual behavior of customers, thus expanding the scope of research. Furthermore, the study relied on self-reported measures to assess individuals’ values, ascribed responsibility, environmental concern, and personal norms, we acknowledge the potential for response error due to the social desirability effect. Therefore, future studies require additional subjective measures, such as peer reports, to counteract these biases. In the future, it is recommended to evaluate the applicability of our theoretical model in different pro-environmental contexts.

## Data availability statement

The raw data supporting the conclusions of this article will be made available by the authors, without undue reservation.

## Ethics statement

Ethical review and approval was not required for the study on human participants in accordance with the local legislation and institutional requirements. The participants provided written informed consent.

## Author contributions

ZD: Conceptualization, Data curation, Formal analysis, Project administration, Writing – original draft. CH: Conceptualization, Investigation, Methodology, Supervision, Writing – original draft, Writing – review & editing. TH: Methodology, Software, Validation, Writing – original draft. TJ: Data curation, Investigation, Methodology, Software, Writing – review & editing.
